# Amino Acylguanidines as Bioinspired Catalysts for the Asymmetric Aldol Reaction

**DOI:** 10.3390/molecules26040826

**Published:** 2021-02-05

**Authors:** Ciril Jimeno

**Affiliations:** Department of Biological Chemistry, Institute of Advanced Chemistry of Catalonia (IQAC-CSIC), Jordi Girona 18-26, E08034 Barcelona, Spain; ciril.jimeno@iqac.csic.es

**Keywords:** guanidines, bioinspired catalysts, aldol reaction, hydroxyacetone, asymmetric catalysis, organocatalysis

## Abstract

The binding and stabilizing effect of arginine residues in certain aldolases served as inspiring source for the development of a family of amino acylguanidine organocatalysts. Screening and optimization led to identify the threonine derivative as the most suitable catalyst for the asymmetric aldol addition of hydroxyacetone, affording the *syn* diastereomer in high ee. In contrast, the proline derivative yielded the *anti* diasteromer. MMFF models suggest the presence of an extensive hydrogen bonding network between the acylguanidinium group and the reaction intermediates.

## 1. Introduction

**Figure 1:**
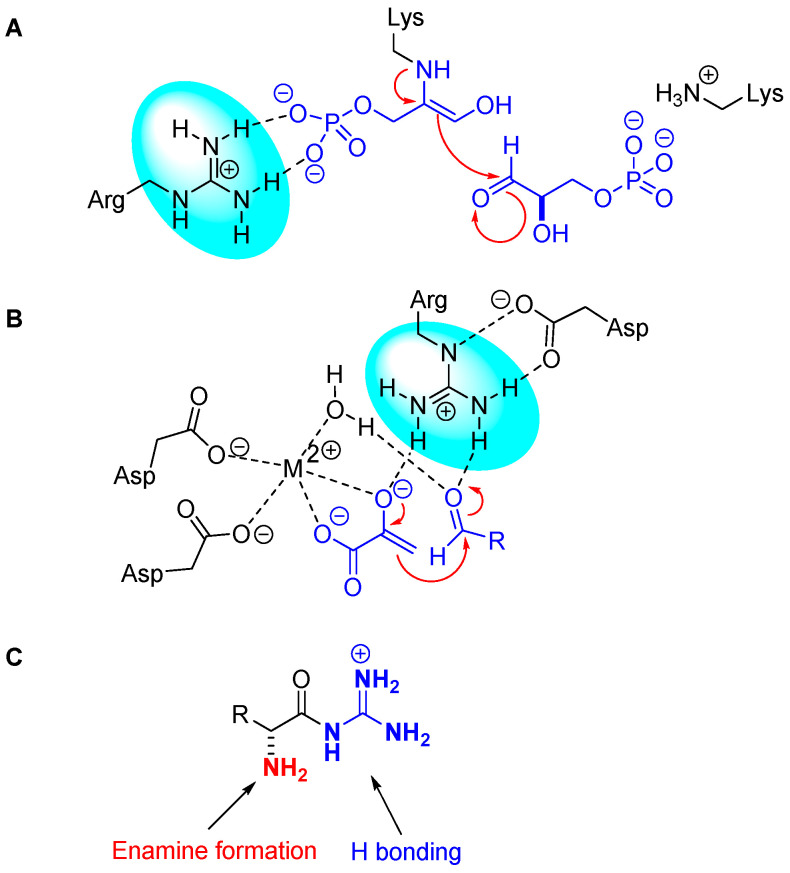
Mechanism of class I fructose bis(phosphate) aldolase (**A**) and class II pyruvate aldolase (**B**). Bioinspired amino acid derived acylguanidinium organocatalysts (**C**).

Aldolases can proceed through two general mechanisms: In class I aldolases, a lysine residue is in charge of the enamine formation with the substrate, which is then able to react with an aldehyde. In contrast, in class II aldolases, substrate enolization takes place through binding to a divalent cation that acts as Lewis acid. Moreover, numerous electrostatic and hydrogen bonding interactions with other residues contribute to the reaction outcome in both reaction mechanisms. In particular, an arginine residue is an additional important parameter in both aldolase classes, participating in the stabilization and orientation of the intermediates through its guanidine group [1,2,3]. This feature made me think that amino guanidines, and in particular, amino acid derived acylguanidines, could become convenient organocatalysts for the asymmetric aldol reaction, exploiting the enamine formation ability of class I aldolases and the intermediates stabilization ability provided by the arginine residue in some class I and II aldolases (Figure 1A,B).

Chiral guanidines have been used successfully for some time as asymmetric organocatalysts [4,5]. Indeed, during our ongoing program on supramolecular and bioinspired organocatalysts [6,7,8], we already developed an *N*-alkyl acylguanidine able to operate in water under emulsion conditions. Remarkable rate acceleration and stereoselectivity were obtained for the aldol reaction of cyclohexanone and aromatic aldehydes [9].

In this communication, a novel family of bioinspired organocatalysts is presented [10] (Figure 1C) based on the hydrogen bonding features of the acylguanidinium group and its application in the asymmetric aldol reaction of hydroxyacetone, a typical substrate for class I aldolases and thoroughly studied in bioinspired catalysis and organocatalysis. Selected examples are given in references [11,12,13,14,15,16,17,18,19,20,21,22,23,24,25,26,27,28,29].

## 2. Results and Discussion

**Scheme 1:**
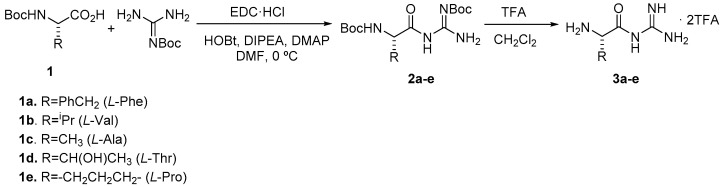
Synthesis of amino acid acylguanidines **3a**–**e**.

Having in mind that the new catalysts were intended to work under homogeneous conditions, a series of *N*-Boc-amino acids were derivatized to acylguanidines **3a**–**e** by coupling with *N*-Boc guanidine and subsequent deprotection with trifluoroacetic acid (Scheme 1). Yields in the range 66–86% were obtained for the coupling reaction after purification by flash chromatography, whereas quantitative yields were found in the deprotection step. Details are given in the Supporting Information.

It is worth mentioning that final catalysts **3a**–**e** were obtained as trifluoroacetate salts. In particular, 2.2–2.4 equivalents of TFA were present in respect to the acylguanidine, as determined by ^1^H/^19^F NMR using 2,2,2-trifluoroethanol as internal standard. This result indicates that **3a**–**e** exist as dications, and that both the amine and the guanidine group are protonated. Indeed, ^1^H-NMR spectra of **3a**–**e** in CD_3_CN show two wide singlets downfield (typically between 7.5–9.5 ppm) integrating 2 H each that are assigned to the protonated N–C(NH_2_)=NH_2_^+^ guanidinium group. On the other hand, the amide N-H and the protonated amino group are difficult to detect as they appear together as very broad signals of low intensity. This behavior indicates fast exchange at the NMR time scale, and therefore higher acidity than the guanidium group.

This set of organocatalysts was subsequently tested in the asymmetric aldol reaction of hydroxyacetone and *p*-nitrobenzaldehyde. Initial screening conditions involved the use of THF as solvent and 10 mol% catalyst loading at 0 °C (Table 1). The Phe derivative **3a** yielded the highest conversion towards product (53% conv.) with moderate enantioselectivities for the two aldol diastereomers (entry 1). The Val and Ala derivatives **3b** and **3c** gave lower conversion but higher enantioselectivity for the aldol reaction: up to 72% ee was obtained for the *syn* aldol with **3b** and 68% ee with **3c** (entries 3 and 5). In turn, the Thr organocatalyst **3d** provided the lowest conversion, just 17%, but the highest ee (82% ee) for the *syn* diastereomer (entry 7). It must be noted that the *syn* diastereomer predominates in the reactions catalyzed by these four organocatalysts, although in a moderate 2 to 1 ratio, approximately. Finally, the Pro acylguanidine **3e** catalyst provided a 31% conversion and 62% ee for the *anti* aldol product, which predominated in this case at a 2.5 to 1 ratio over the *syn* diastereomer (Entry 9) (Table 1).

The reaction was repeated by letting them warm up slowly from 0 °C to rt. Yields effectively increased this time in all cases (Entries 2, 4, 6, 8, and 10, Table 1), but stereoselectivity did not significantly improve except for two cases: for catalyst **3d**, dr increased from 1/2.5 to 1/3.8 (*anti/syn*) although ee stayed the same (Entry 8). For catalyst **3e**, dr increased a little bit, as well as the ee (Entry 10, Table 1). Similar reaction conditions but adding NaHCO_3_ to obtain the free amino groups of catalysts **3a**–**e** at rt only produced deleterious results (See Supplementary).

Given the important solvent effects commonly observed in organocatalysis, solvent was optimized next. The Thr derivative **3d**, which gave the highest stereoselectivity before, was used. We expected to increase ee to >90% ee along a significant rate acceleration due to the solvent (Table 2).

Unfortunately, with these results in hand, it was clear that the expected concomitant rate acceleration and stereoselectivity increase was not achieved. However, excellent ee and dr for the *syn* diastereomer was found with DMF (Entry 2, Table 2), only approached by results obtained in ethanol.

In the next step, the concentration was optimized. We have showed recently how dilution was an important parameter to reach actually higher reaction rates and enantioselectivity in organocatayzed Michael additions [30,31]. In the current case, results are summarized in Table 3. Reactions were carried out at rt for 24 h, using this time a 20 mol% catalyst loading to further favor reaction acceleration.

In terms of stereoselectivity, best results were obtained under diluted conditions (Entry 1, Table 3), which allowed the aldol reaction to proceed with an *anti/syn* dr of 1/4.4 and 91% ee. Upon concentration, stereoselectivity slowly eroded even though conversion increased a bit (Compare entries 1 to 3). Finally, under neat conditions, wherein hydroxyacetone in excess acted as the only solvent, conversion did not improve and stereoselectivity was especially poorer (Entry 4). This drawback seems to be due to the absence of DMF. As I have shown in Table 2, the solvent plays a fundamental role in stereoselectivity.

Therefore, to achieve practical conditions with DMF, catalyst loading of **3d** was increased to 20 mol% at the same time that the reaction was carried out entirely at rt while increasing reaction time to 2 days. In this way, high conversion and stereoselectivity were eventually observed (Table 4, Entry 1). It must be noted that results at 0 °C implied only a slight increase in ee, whereas a sharp fall in conversion was observed (Entry 2).

Other aldehydes with electron withdrawing groups were tested as well: the 2,4-dichloro derivative showed excellent conversion (99%), diastereoselectivity (*anti/syn* 1/12) and enantiomeric excess (94% ee), whereas o-fluorobenzaldehyde gave lower yield (64% conversion) but still excellent enanioselectivity (97% ee for the *syn* diastereomer, See Entries 3 and 4, Table 4). The less electron withdrawing m-chlorobenzaldehyde, however, yielded lower conversion after three days of reaction, although still with good levels of stereoselectivity (58% conversion and 89% ee, entry 5, Table 4). In turn, benzaldehyde, which is even less reactive, gave a sluggish reaction with only 18% conversion after 4 days and poorer ee (86% ee) (Entry 6). This trend of reactivity and enantioselectivity was already noted by us for hydrophobic acylguanidines operating in aqueous medium [9].

Since the Pro derivative **3e** yielded the *anti* aldol diastereomer, it was tested again under the new conditions (20 mol%, DMF at rt for 2 days). Excellent yield but moderate stereoselectivity were observed, in any case lower than that of the threonine derivative **3d** (Entry 7, Table 4).

To assess the functionality of the acylguanidine group, the benchmark asymmetric aldol reaction was carried out using the parent free amino acids as catalysts under identical reaction conditions. L-proline yielded lower conversion and regioselectivity than **3e** (76% conversion taking into account 18% of the *iso* regioisomer vs. 95% conv.) but higher enantiomeric excess (92% vs. 77% ee, entries 7 and 8). In contrast, reaction with L-threonine was sluggish, with just 11% conversion after 2 days compared to 94% conversion for **3d**. Moreover, enantioselectivity was also much poorer for l-Thr, affording 69% ee as compared to 91% ee for **3d** (Entries 1 and 9, Table 4). Therefore, the acylguanidinium group provides a convenient enhancement of the catalytic characteristics of threonine, although this is not so clear for proline, at least in terms of ee.

**Figure 2:**
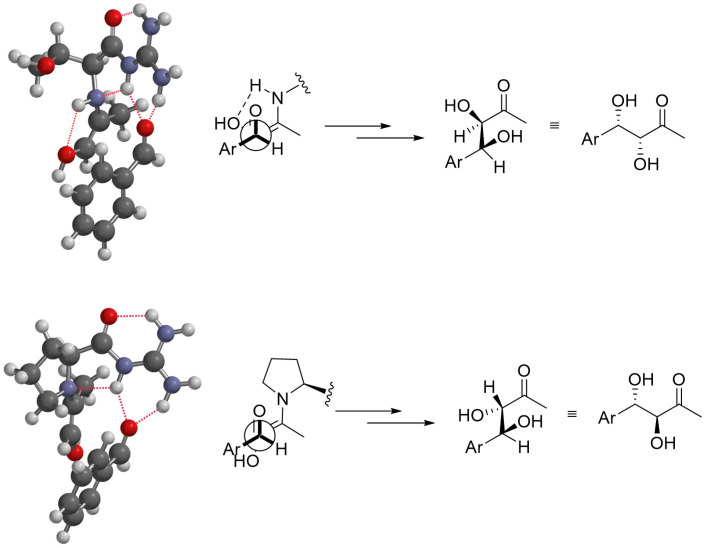
MMFF models for the asymmetric addition of hydroxyacetone to *p*-nitrobenzandehyde catalyzed by **3d** (**top**) and **3e** (**bottom**). Hydrogen bonding showed as red dotted lines.

Finally, a few words on the absolute configuration of the aldol products: the absolute configuration of the major *syn* diastereomer obtained with catalyst **3d** was determined by comparison of its HPLC trace with that of the product arising from asymmetric dihydroxylation of (*E*)-4-(4-nitrophenyl)but-3-en-2-one using Sharpless’ AD mix α [25,32]. It corresponds to (3*R*,4*S*), as depicted throughout this paper. It must be noted that the *anti* diastereomer has opposite configuration depending on whether the catalyst is **3a**–**e** or **3d**. The *anti* configuration shown throughout this paper corresponds to the major enantiomer of the *anti* diastereomer obtained with catalysts **3a**–**e**. Therefore, the major compound obtained with proline derivative **3d** corresponds to the (3*S*,4*S*) isomer (See Figure 2 bottom). The HPLC traces and the assignment of configuration are shown in the Supplementary Materials section.

Indeed, the absolute configuration of the predominant products match those obtained through established models for these reactions [23,25]. In Figure 2, the molecular mechanics models for reactions catalyzed by **3d** and **3e** (not transition states) are shown. Interestingly, a stabilizing hydrogen bonding network amongst the acylguanidinium moiety, the enamine *N* and the aldehyde carbonyl *O* seems to be an important feature of these catalysts [33]. However, the exact stabilizing effect is not known, since it would require high level computational studies out of the scope of this communication.

## 3. Conclusions

In conclusion, a novel family of amino acid derived acylguanidines for the asymmetric aldol reaction of hydroxyacetone have been synthesized, characterized, and optimized. These compounds were isolated as diprotonated species (ammonium-guanididium) and the threonine derivative **3d** afforded high levels of stereoselectivity for the *syn* aldol diasteromer, surpassing the characteristics of free threonine. In contrast, the proline derivative **3e** afforded the *anti* diastereomer, but with less practical results. The operation of these catalysts was inspired by the mode of action of some natural aldolases wherein arginine residues participate in the stabilization of intermediates through hydrogen bonding. Actually, simple molecular mechanics models do suggest the presence of a widespread hydrogen bonding network amongst the acylguanidinium moiety and the reaction intermediates that would be responsible for the observed reactivity and stereoselectivity. Further applications of this class of organocatalysts are under investigation and will be reported at due course.

## Figures and Tables

**Table 1:**
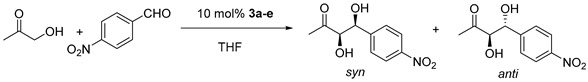
Screening of acylguanidines **3a**–**e** in the asymmetric aldol reaction.

Entry.	Catalyst	Conversion/% ^c^	d.r. *anti/syn* ^c^	ee *anti*/% ^d^	ee *syn*/% ^d^
1	**3a**	53 ^a^	1/1.9 ^a^	50 ^a^	55 ^a^
2		70 ^b^	1/2 ^b^	37 ^b^	40 ^b^
3	**3b**	27 ^a^	1/1.4 ^a^	59 ^a^	72 ^a^
4		43 ^b^	1/2 ^b^	41 ^b^	68 ^b^
5	**3c**	25 ^a^	1/2.3 ^a^	43 ^a^	60 ^a^
6		44 ^b^	1/2.3 ^b^	45 ^b^	48 ^b^
7	**3d**	17 ^a^	1/2.5 ^a^	52 ^a^	82 ^a^
8		33 ^b^	1/3.8 ^b^	51 ^b^	81 ^b^
9	**3e**	31 ^a^	2.5/1 ^a^	62 ^a^	35 ^a^
10		46 ^b^	2.7/1 ^b^	69 ^b^	41 ^b^

^a^ Performed at 0 °C for 24 h. ^b^ Performed from 0 °C to rt, for 24 h. ^c^ Determined by ^1^H NMR. ^d^ Determined by HPLC on a chiral stationary phase (ID column).

**Table 2:**
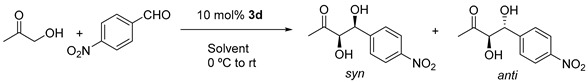
Effect of solvent in the asymmetric aldol reaction catalyzed by **3d**.

Entry	Solvent	Conversion/% ^c^	d.r. *anti/syn* ^c^	ee *syn*/% ^d^
1	THF	33	1/3.8	81
2	DMF	19	1/5.0	92
3	CH_2_Cl_2_^a^	3	1/1.4	57
4	CH_3_CN	41	1/2.3	80
5	AcOEt ^b^	46	1/2.0	67
6	^i^PrOH ^b^	59	1/2.0	79
7	EtOH	23	1/2.8	89
8	water	N. R.	-	-

^a^ 15 mol% catalyst loading. ^b^ 20 mol% catalyst loading. ^c^ Determined by ^1^H NMR. ^d^ Determined by HPLC on a chiral stationary phase (ID column). N. R. No reaction.

**Table 3:**
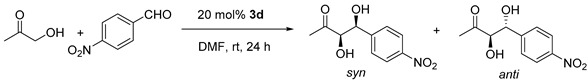
Effect of concentration in the aldol reaction catalyzed by **3d**.

Entry	Conc./M ^a^	Conv./% ^c^	dr *anti/syn* ^c^	ee *syn*/% ^d^
1	0.18	75	1/4.4	91
2	0.31	75	1/4.1	90
3	0.46	81	1/3.7	88
4	1.43 ^b^	71	1/1.6	57

^a^*p*-Nitrobenzaldehyde concentration. ^b^ Neat (No DMF). ^c^ Determined by ^1^H NMR. ^d^ Determined by HPLC on a chiral stationary phase (ID column).

**Table 4:**
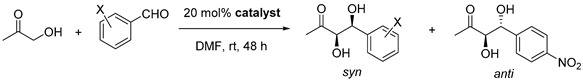
Optimized conditions for the asymmetric aldol reaction.

Entry	Catalyst	Aldehyde	Conversion/% ^d^	d.r. *anti/syn* ^d^	ee Major/% ^e^
1	**3d**	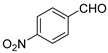	94	1/4.5	91
2			26 ^a^	1/4.4 ^a^	93 ^a^
3		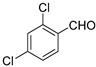	99	1/12	94
4		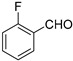	64	1/4.2	97
5 ^b^		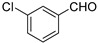	58 ^b^	1/4.5 ^b^	89 ^b^
6 ^c^		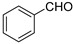	18 ^c^	1/3 ^c^	86 ^c^
7	**3e**	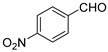	95	3.7/1	77
8	**L-Pro**		76 ^f^	3.1/1	92
9	**L-Thr**		11	1/3.7	69

^a^ Performed at 0 °C. ^b^ 3 days reaction. ^c^ 4 days reaction. ^d^ Determined by ^1^H NMR. ^e^ Determined by HPLC on a chiral stationary phase. ^f^ Comprises 18% of the *iso* regioisomer.

## Supplementary Materials

The following are available online, experimental procedures and characterization data, along with ^1^H and ^13^C NMR spectra of catalysts **3a**–**e**. Chiral HPLC traces.
