# Supplementary material for: Amino Acylguanidines as Bioinspired Catalysts for the Asymmetric Aldol Reaction
Source: Molecules. 2021 Feb 5;26(4):826. doi: 10.3390/molecules26040826 (PMC7915246; doi:10.3390/molecules26040826)
Supplement: Supplementary file 1 [file molecules-26-00826-s001.pdf]

SUPPORTING INFORMATION

ACYLGUANIDINES DERIVED FROM AMINO ACIDS AS BIOINSPIRED ASYMMETRIC ORGANOCATALYSTS

Ciril Jimeno

Institute of Advanced Chemistry of Catalonia (IQAC-CSIC)

Jordi Girona 18-26

E08034 Barcelona (Spain)

INDEX

1. Synthesis and characterization of catalysts **3a-e**. S3
2. ^{1}H and ^{13}C NMR spectra for 3a-e. S6
3. Procedure for the asymmetric aldol reaction of hydroxyacetone and *p*-nitrobenzaldehyde. S11
4. Asymmetric aldol reaction of hydroxyacetone in the presence of $NaHCO_3$. S11
5. HPLC traces and absolute configuration determination of 3,4-dihydroxy-4-(4-nitrophenyl)butan-2-one. S12
6. d.r. determination for 3,4-dihydroxy-4-(4-nitrophenyl)butan-2-one. S13
7. ee and d.r. determination for additional compounds in table 4. S14

1. SYNTHESIS AND CHARACTERIZATION OF CATALYSTS **3a-e**:

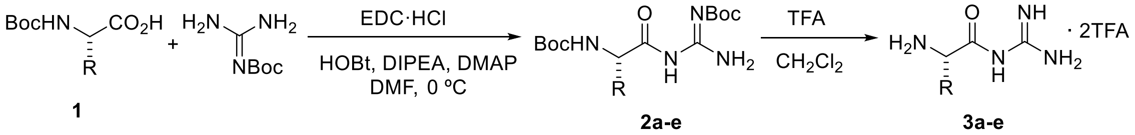

1a. R=$PhCH_2$ (*L*-Phe)
1b. R=^iPr (*L*-Val)
1c. R=CH_3 (*L*-Ala)
1d. R=$CH(OH)CH_3$ (*L*-Thr)
1e. R=-$CH_2CH_2CH_2$- (*L*-Pro)

Typical procedure: synthesis of L-phenylglycine acylguanidine 3a:

To a solution of L-Boc-Phe (1.0 g, 3.77 mmol) in 38 ml of dry DMF at 0 ºC were successively added EDC·HCl (795 mg, 4.15 mmol), HOBt (764 mg, 5.66 mmol), DIPEA (722 μL, 4.15 mmol) and DMAP (46 mg, 0.38 mmol). After 1 h, Boc-guanidine (0.90 g, 5.65 mmol) was added and the resulting mixture was stirred at rt overnight.

Afterwards, the reaction mixture was diluted with AcOEt (150 mL) and washed with aq. sat. $NaHCO_3$ (2x30 mL) and brine (2x30 mL). The organic layer was dried over $MgSO_4$ and evaporated in the rotavap. The crude product was purified by flash chromatography eluting with dichloromethane:methanol mixtures from 100:0 to 97:3. 1.32 g (86% yield) of **2a** were isolated.

Yields for **2b** (66%), **2c** (74%), **2d** (73%) and **2e** (71%).

Then, 1.20 g (2.95 mmol) of **2a** were dissolved in 18 mL of a 1:2 TFA:CH_2Cl_2 mixture, and stirred at rt for 1 h 45 min. Afterwards, liquids were removed under a N_2 flow and gentle heating. The residue was dissolved in water and freeze dried.

All yields for compounds **3a-3e** were quantitative, having a slight excess of TFA (2.2-2.4 equivalents) as determined by $^1H/^{19}F$ NMR using 2,2,2-trifluoroethanol as internal standard.

3a·2TFA, (S)-2-amino-N-carbamimidoyl-3-phenylpropanamide (L-phenylalanine acylguanidine) bis(trifluoroacetic acid) salt:

^1H-NMR (400 MHz, CD_3CN): δ 3.04 (dd, *J*=14.4, 9.7 Hz, 1H), 3.40 (dd, *J*=14.4, 4.3 Hz, 1H), 4.38 (dd, *J*= 9.7, 4.4 Hz, 1H), 7.37 (m, 5H), 8.74 (s, 2H), 9.11 (s, 2H) ppm.

$^{13}C\{^1H\}$-NMR (100 MHz, CD_3CN): δ 37.2 (CH_2), 57.2 (CH), 129.0 (C), 130.0 (CH), 130.7 (CH), 134.6 (C), 157.1 (C), 172.3 (C) ppm.

$^{19}F\{^1H\}$-NMR (376 MHz, CD_3CN): δ -76.3 ppm

MS (ESI-TOF+): m/z 120 (100%, $PhCH_2CH_2NH_2^+$), 207 (80%, $M+H^+$), 413 (25%, $2M+H^+$)

HRMS (ESI-TOF+): m/z 207.1328 (Calculated for $C_{10}H_{15}N_4O^+$: 207,1240)

$[\alpha]_D$ (c 1.0, MeOH): +23.5

3b·2TFA, (S)-2-amino-N-carbamimidoyl-3-methylbutanamide (L-valine acylguanidine) bis(trifluoroacetic acid) salt:

^{1}H-NMR (400 MHz, CD_3CN): δ 0.97 (d, *J*=6.9 Hz, 3H), 1.07 (d, *J*=6.9 Hz, 3H), 2.34 (m, 1H), 4.09 (d, *J*=4.5 Hz, 1H), 7.9 (s, 2H), 9.07 (s, 2H) ppm.

^{13}C{^{1}H}-NMR (100 MHz, CD_3CN): δ 16.9 (CH_3), 18.7 (CH_3), 30.7 (CH), 60.7 (CH), 156.7 (C), 172.3 (C) ppm.

^{19}F{^{1}H}-NMR (376 MHz, CD_3CN): δ -71.3 ppm

MS (ESI-TOF+): m/z 159 (95%, M+H^{+}), 317 (100%, 2M+H^{+})

HRMS (ESI-TOF+): m/z 159.1352 (Calculated for $C_6H_{15}N_4O^+$: 159,1240)

[α]$_{405\ nm}$: +5.2 (c=1.4, MeOH)

3c·2TFA, (S)-2-amino-N-carbamimidoylpropanamide (L-alanine acylguanidine) bis(trifluoroacetic acid) salt:

^{1}H-NMR (400 MHz, CD_3CN): δ 1.56 (d, 3H), 4.22 (q, 1H), 7,09 (bs, 4H), 8.11 (s, 2H), 9.08 (s, 2H) ppm.

^{13}C{^{1}H}-NMR (100 MHz, CD_3CN): δ 16.6 (CH_3), 51.8 (CH), 157.0 (C), 173.3 (C) ppm.

^{19}F{^{1}H}-NMR (376 MHz, CD_3CN): δ -71.2 ppm

MS (ESI-TOF+): m/z 131 (45%, M+H^{+}), 261 (100%, 2M+H^{+})

HRMS (ESI-TOF+): m/z 131.1084 (Calculated for $C_4H_{11}N_4O$+: 131,0927)

[α]$_D$ (c 1.1, MeOH): -10.3

3d·2TFA, (2S,3R)-2-amino-N-carbamimidoyl-3-hydroxybutanamide (L-threonine acylguanidine) bis(trifluoroacetic acid) salt:

^{1}H-NMR (400 MHz, CD_3CN): δ 1.29 (2, *J*=6.5 Hz, 3H), 4.11 (d, *J*=3.3 Hz, 1H), 4.30 (m, 1H), 5.52 (bs, 4 H), 8.05 (s, 2H), 9.06 (m, 2H) ppm.

^{13}C{^{1}H}-NMR (100 MHz, CD_3CN): δ 20.1 (CH_3), 60.7 (CH), 66.3 (CH), 156.9 (C), 171.4 (C) ppm.

^{19}F{^{1}H}-NMR (376 MHz, CD_3CN): δ -76.5 ppm

MS (ESI-TOF+): m/z 161 (100%, M+H^{+}), 321 (70%, 2M+H^{+})

HRMS (ESI-TOF+): m/z 161.1148 (Calculated for $C_5H_{13}N_4O_2^+$: 161,1033)

[α]$_D$ (c 1.2, MeOH): -10.8

3e·2TFA, (S)-N-carbamimidoylpyrrolidine-2-carboxamide (L-proline acylguanidine) bis(trifluoroacetic acid) salt:

^{1}H-NMR (400 MHz, CD_3CN): δ 2.02 (m, 2H), 2.13 (m, 1H), 2.48 (m, 1H), 2.80 (bs, 3H), 3.38 (m, 2H), 4.51 (dd, *J*=9.0, 6.7 Hz, 1H), 8.93 (s, 2H), 9.06 (s, 2H) ppm.

^{13}C{^{1}H}-NMR (100 MHz, CD_3CN): δ 24.8 (CH_2), 30.2 (CH_2), 48.0 (CH_2), 61.8 (CH), 157.2 (C), 172.5 (C) ppm.

$^{19}F\{^{1}H\}$-NMR (376 MHz, CD_3CN): δ -76.2 ppm

MS (ESI-TOF+): m/z

HRMS (ESI-TOF+): m/z 157.1173 (Calculated for $C_6H_{13}N_4O^+$: 157,1084)

$[\alpha]_D$ (c 1.1, MeOH): -41.5

2. ^{1}H and ^{13}C NMR SPECTRA FOR **3a-3e**.

L-phenylalanine acylguanidine bis(trifluoroacetic acid) salt, 3a·2TFA

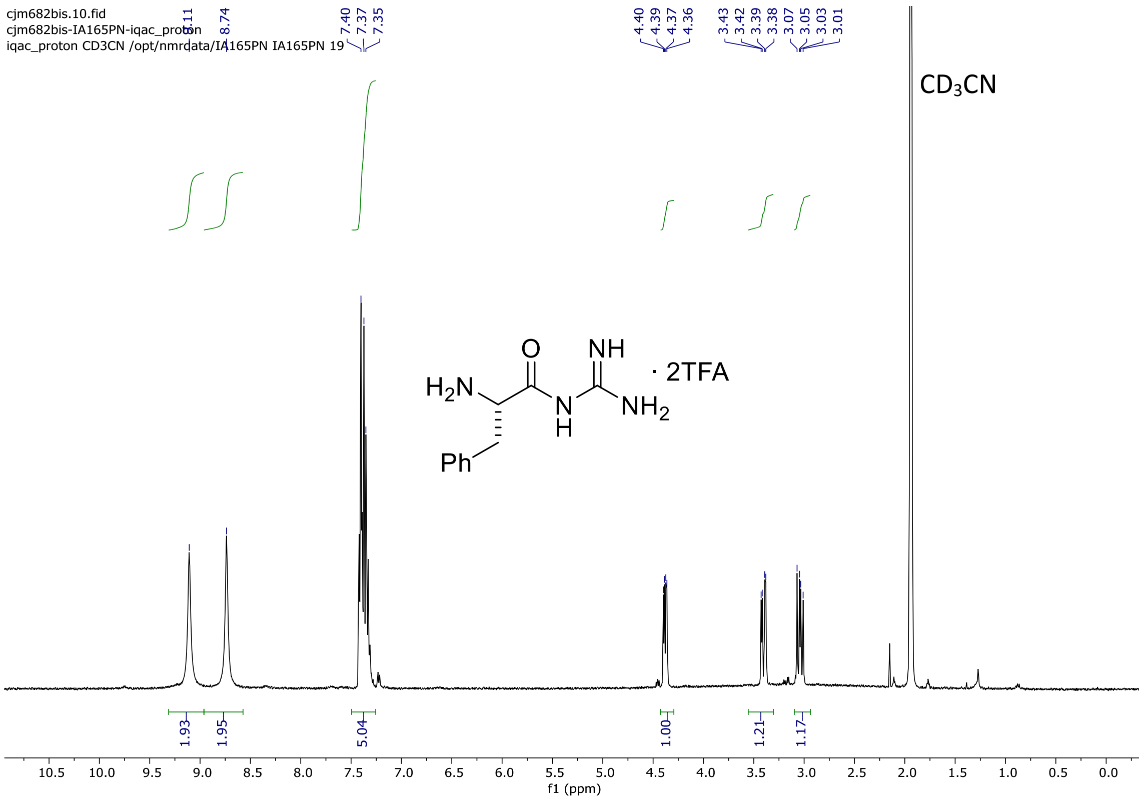

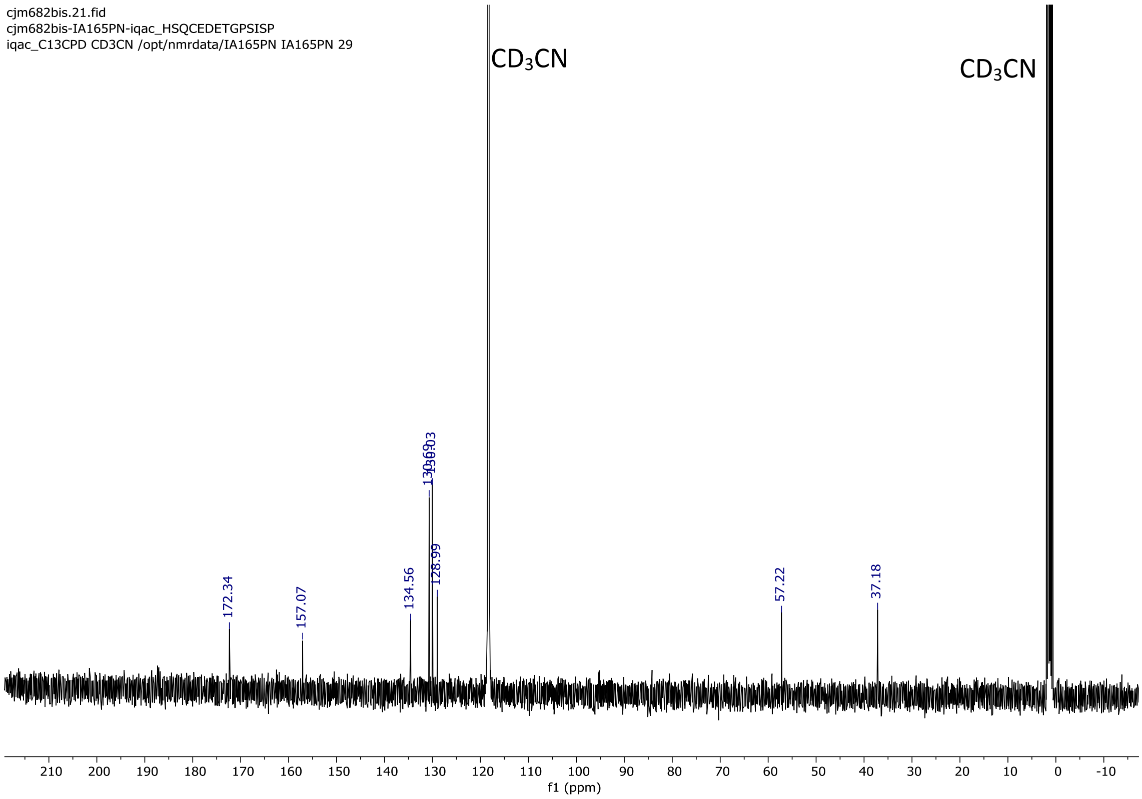

L-valine acylguanidine bis(trifluoroacetic acid) salt, 3b·2TFA

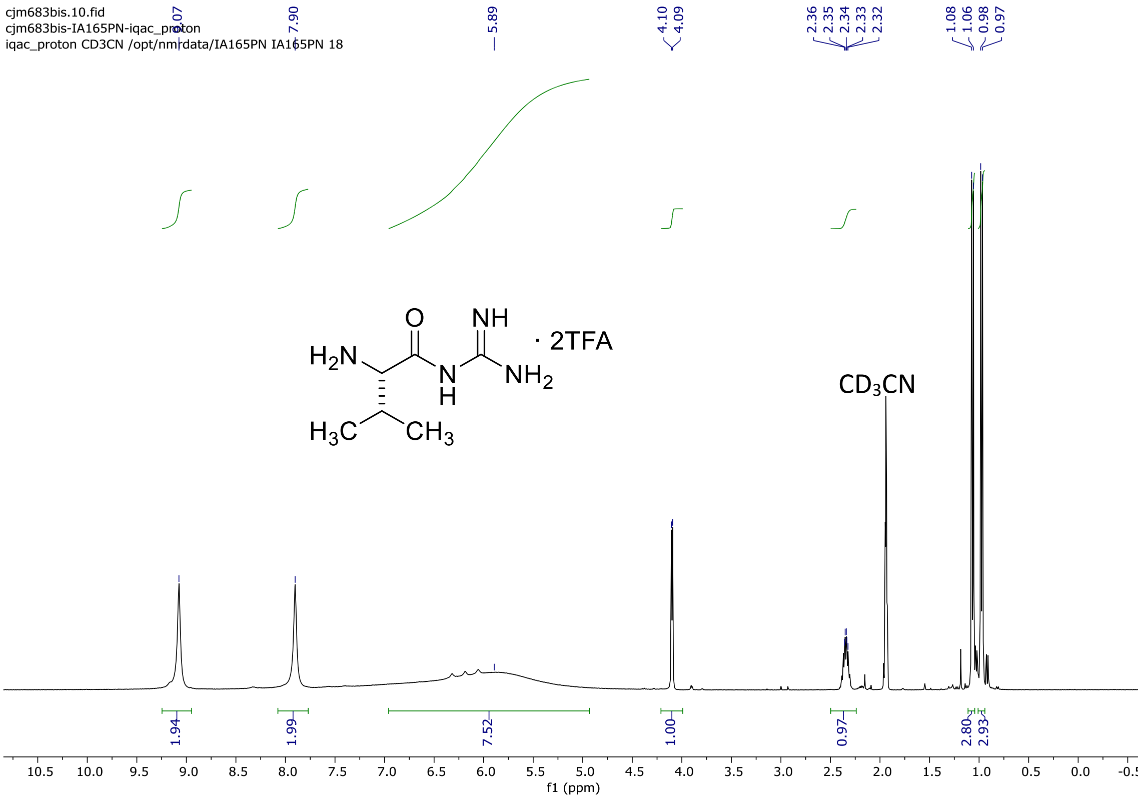

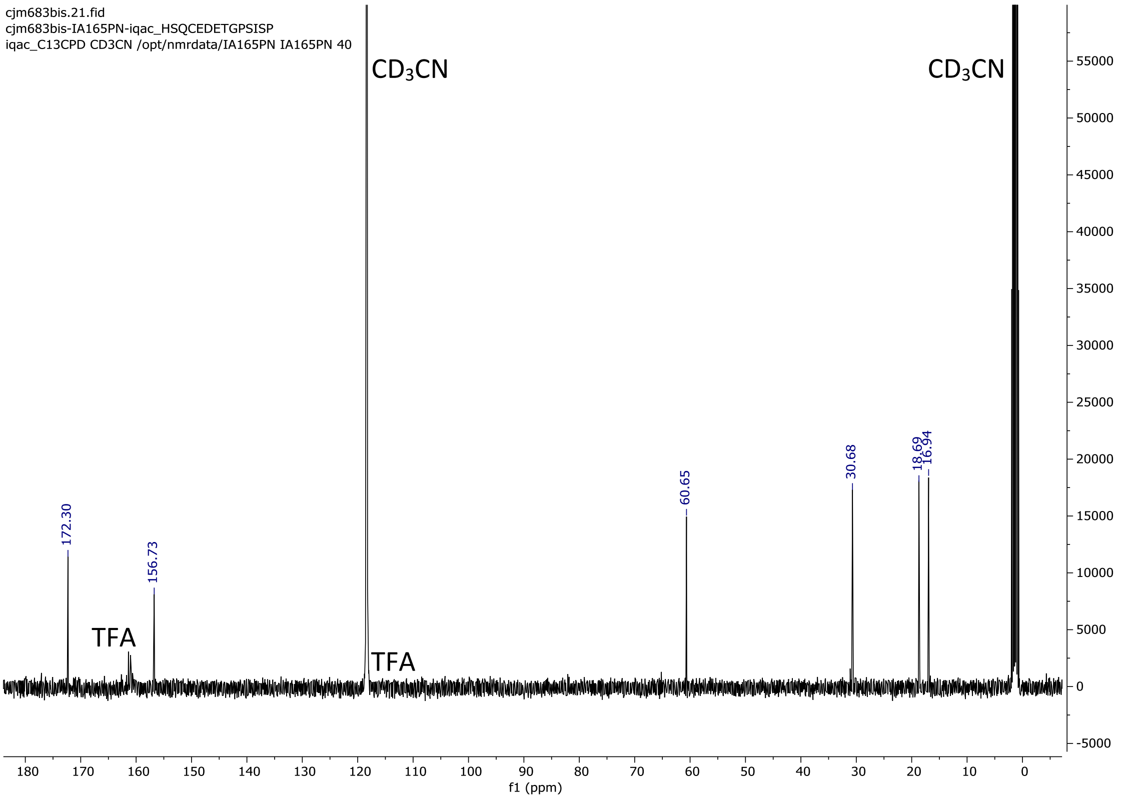

L-alanine acylguanidine bis(trifluoroacetic acid) salt, 3c·2TFA

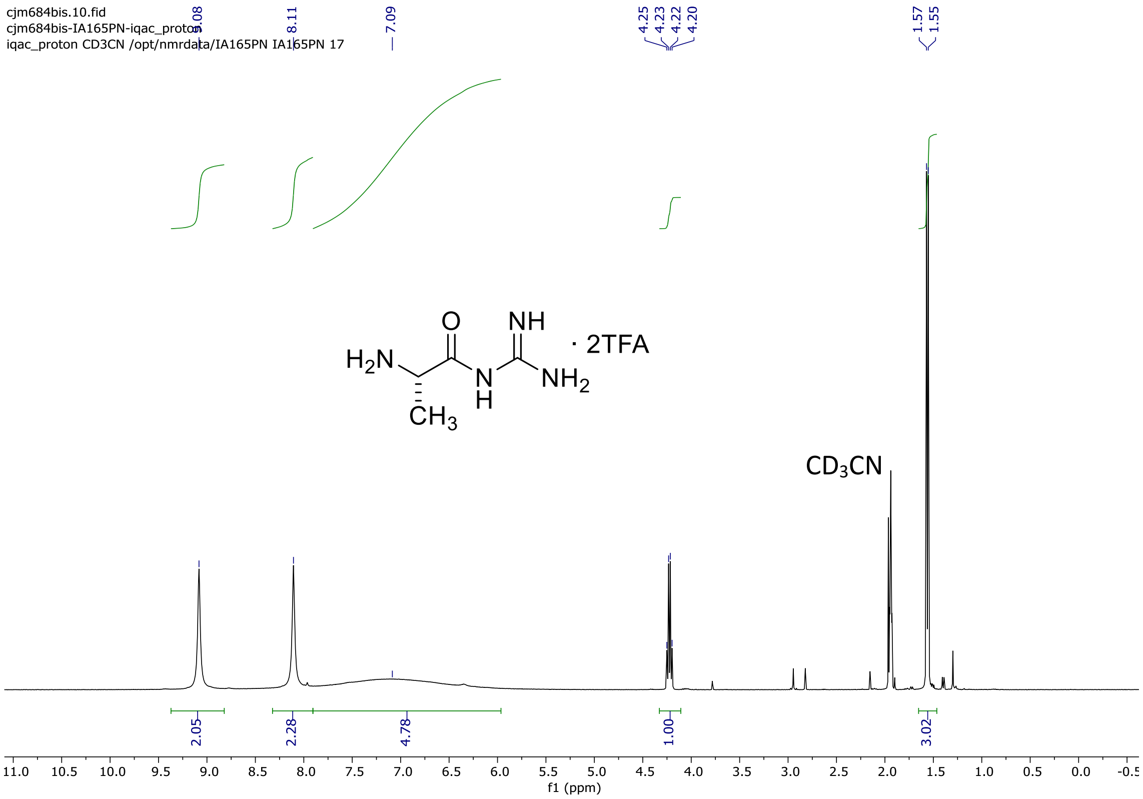

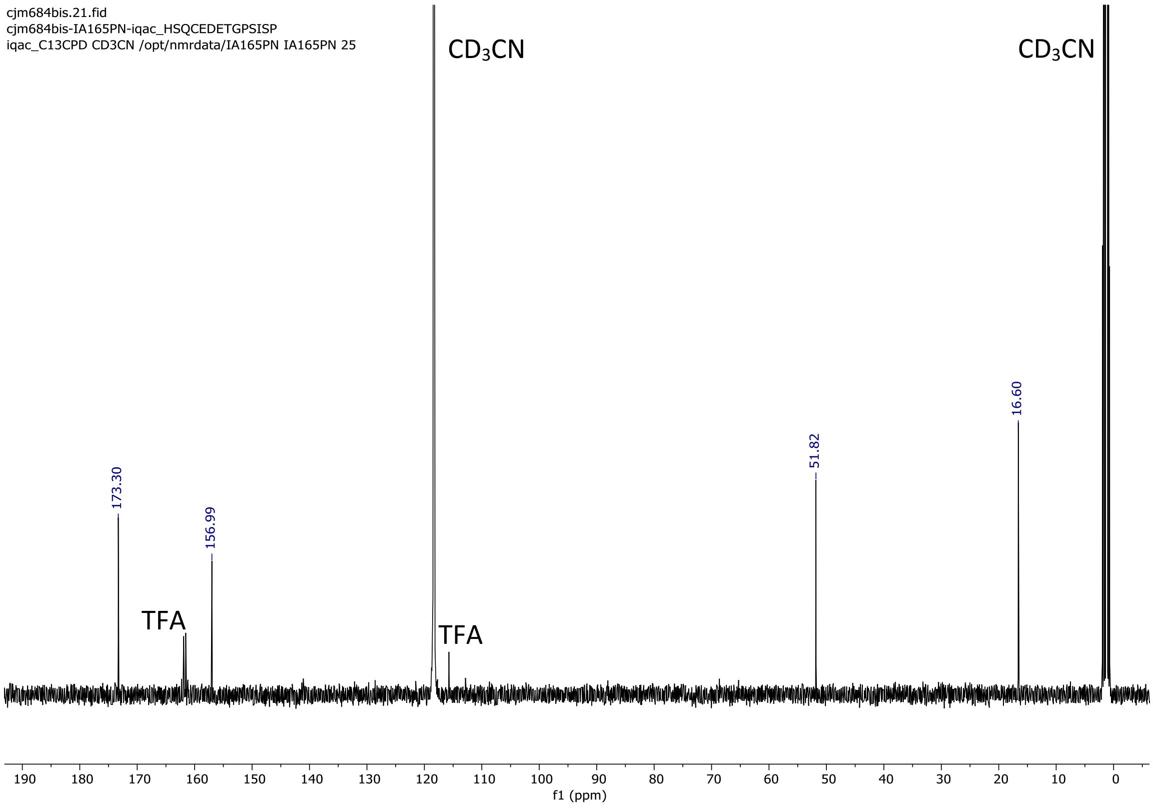

L-threonine acylguanidine) bis(trifluoroacetic acid) salt, 3d·2TFA

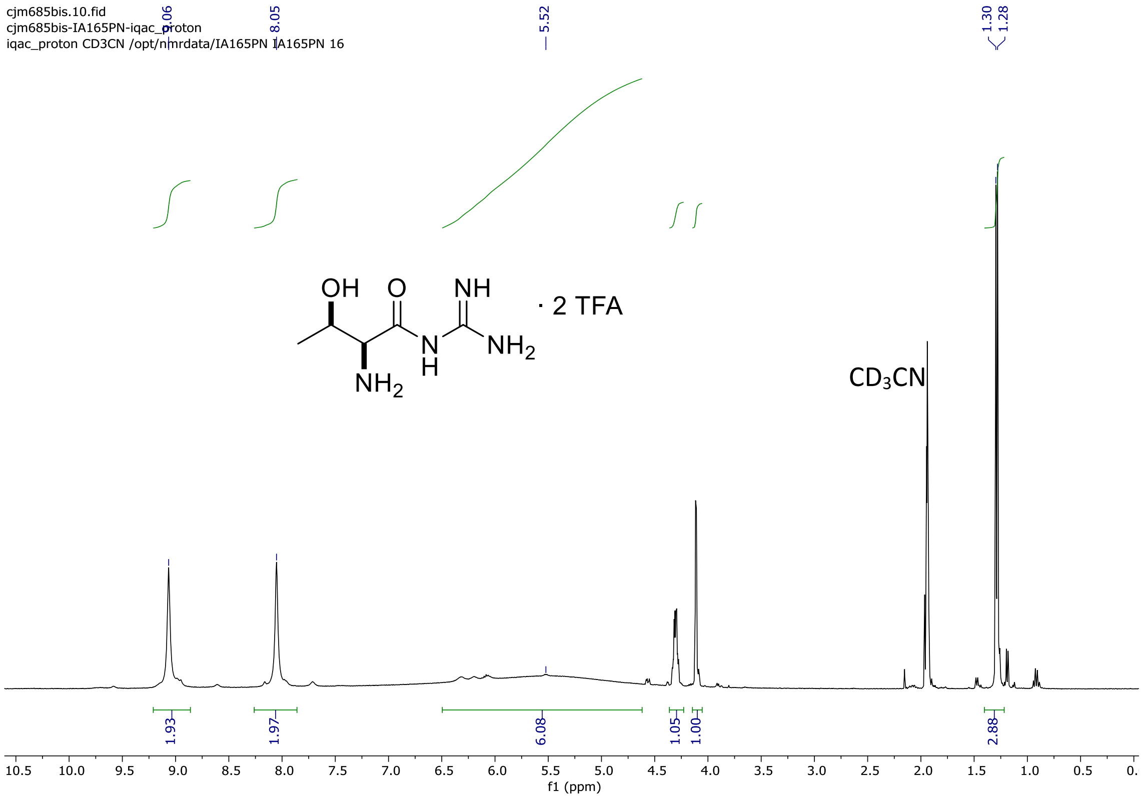

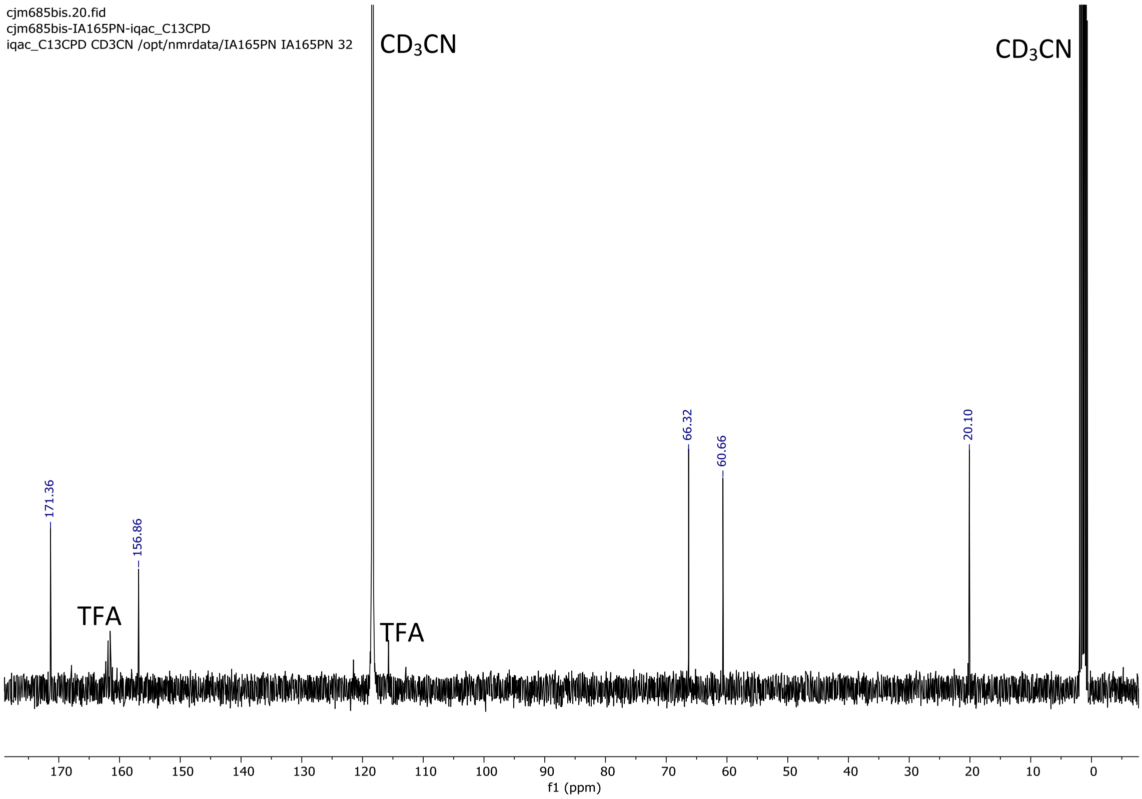

L-proline acylguanidine bis(trifluoroacetic acid) salt, 3e·2TFA

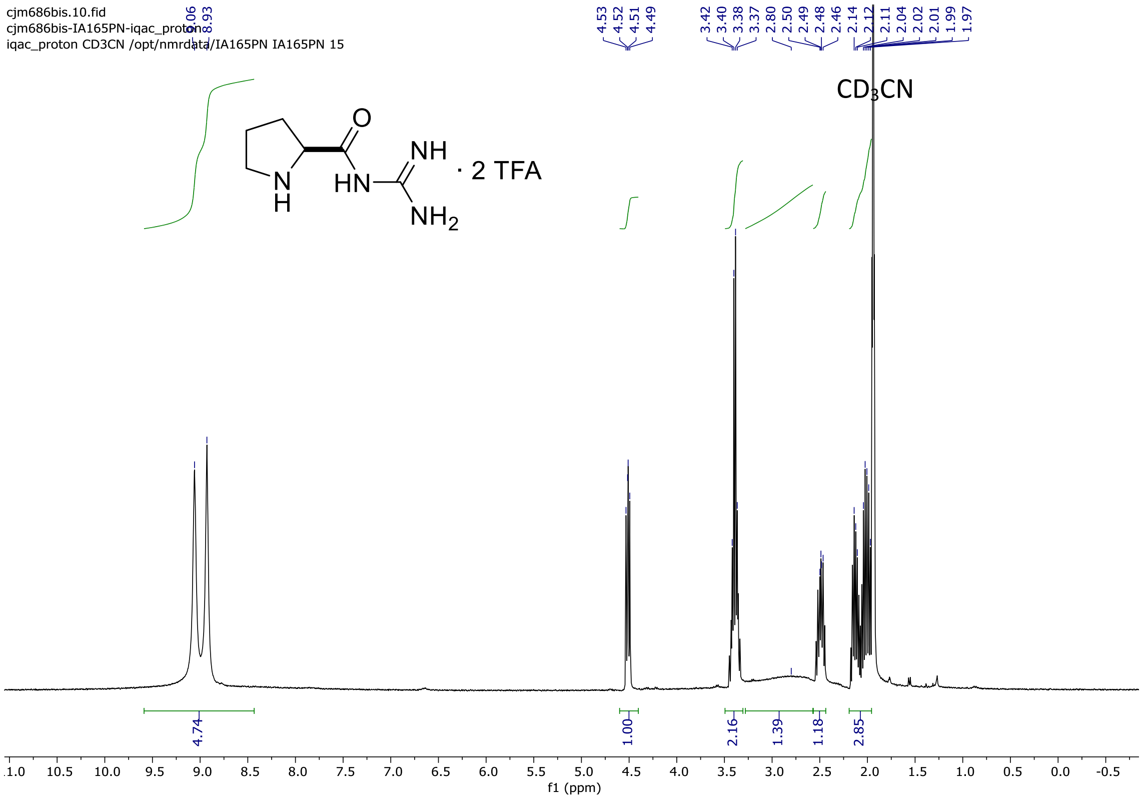

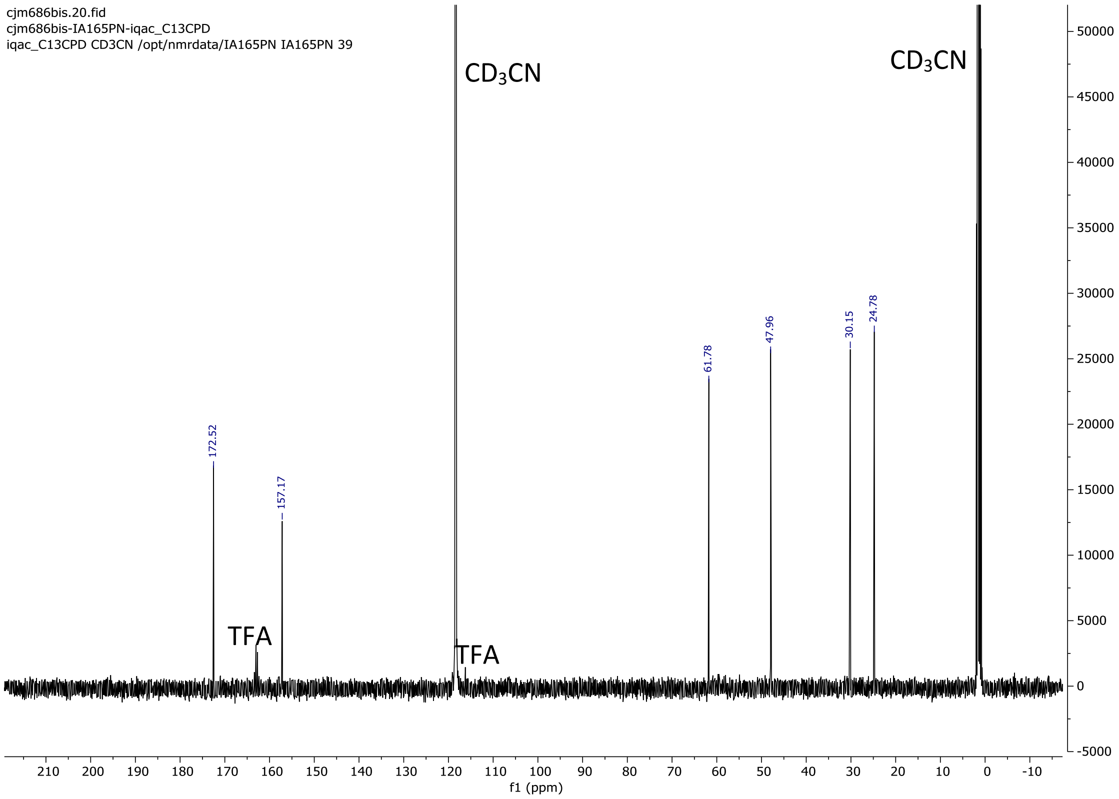

3. PROCEDURE FOR THE ASYMMETRIC ALDOL REACTION OF HYDROXYACETONE AND p-NITROBENZALDEHYDE

38 mg (0.1 mmol) of catalyst 3d and 75 mg (0.5 mmol) of *p*-nitrobenzaldehyde were weighed in a vial at rt. Then, 2.5 ml DMF were added. The mixture was stirred for 5 minutes until complete dissolution of the reactants, and 350 μL (5 mmol) of hydroxyacetone were added. After 48 h, the reaction was quenched with water and extracted with AcOEt (3x10 ml), dried (Na_2SO_4) and solvents removed in the rotavapor. Conversion and disatereoselectivity was determined by ^{1}H NMR on the crude sample. Enantiomeric excess was determined by chiral HPLC (ID, hexane:isopropanol 90:10, 1 ml/min, 254 nm).

4. ASYMMETRIC ALDOL REACTION OF HYDROXYACETONE IN THE PRESENCE OF $NaHCO_3$:

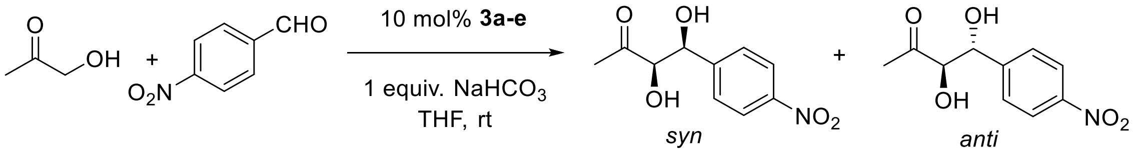

Entry	catalyst	Conv./%	dr *anti/syn*	ee *anti*/%	ee *syn*/%
1	**3a**	31	1/2	30	35
2	**3b**	20	1/1.7	45	58
3	**3c**	27	1/2	21	42
4	**3d**	19	1/2.3	47	74
5	**3e**	60*	2/1	60	14

*90% selectivity (10% of terminal aldol)

5. HPLC TRACES AND ABSOLUTE CONFIGURATION DETERMINATION OF 3,4-DIHYDROXY-4-(4-NITROPHENYL)BUTAN-2-ONE.

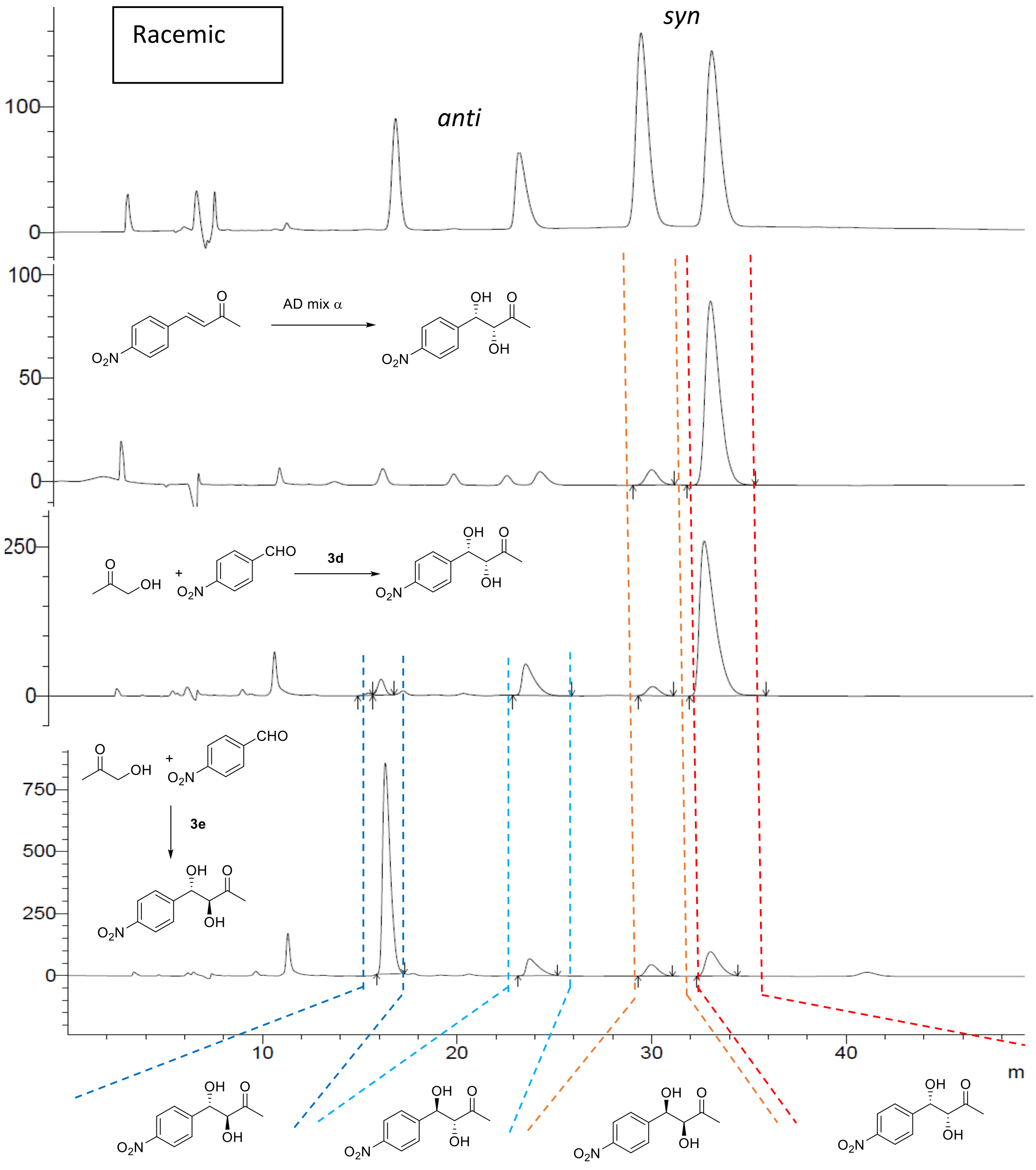

Chiralcel ID column, hexane:isopronanol 90:10, 1 ml/min, 209 nm.

6. d.r. DETERMINATION FOR 3,4-DIHYDROXY-4-(4-NITROPHENYL)BUTAN-2-ONE.

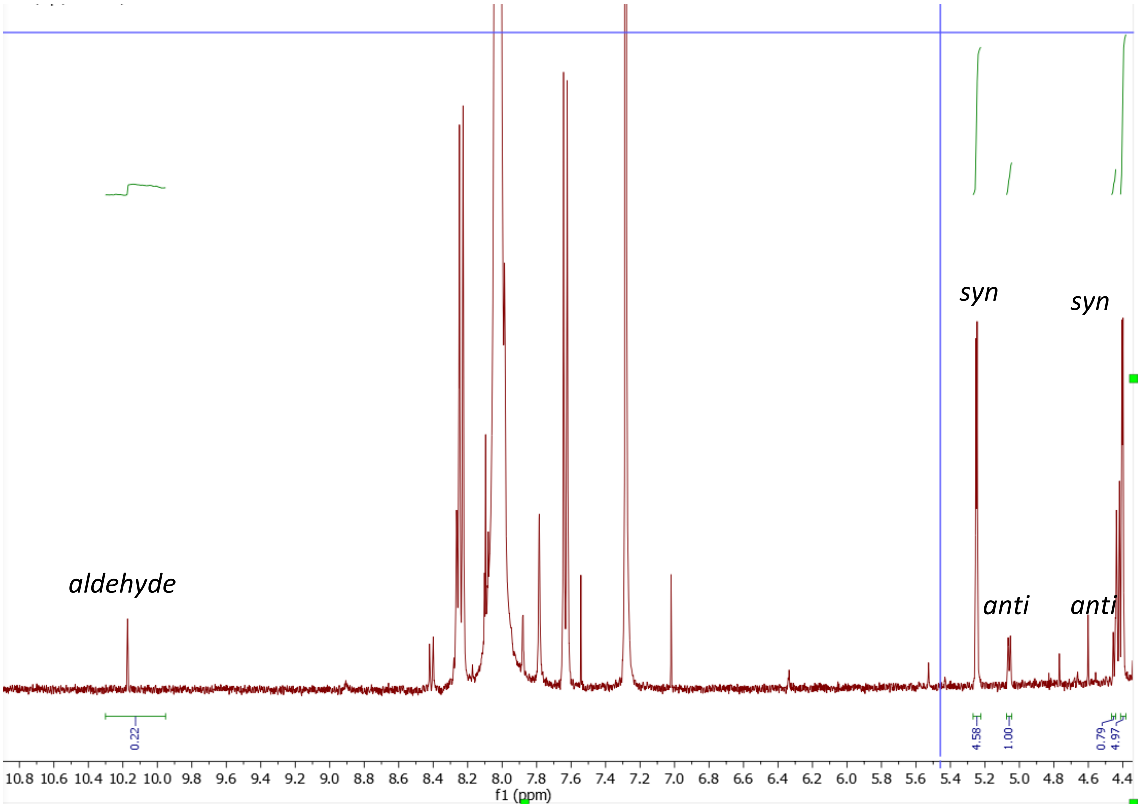

7. <u>ee AND d.r. DETERMINATION FOR ADDITIONAL COMPOUNDS IN TABLE 4.</u>

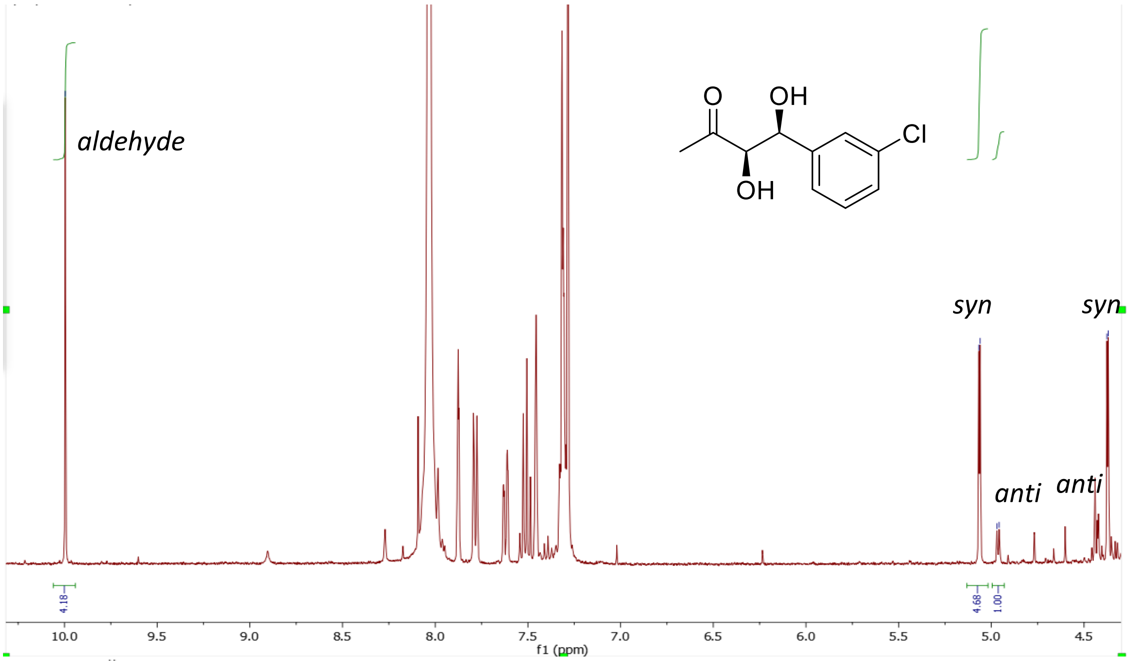

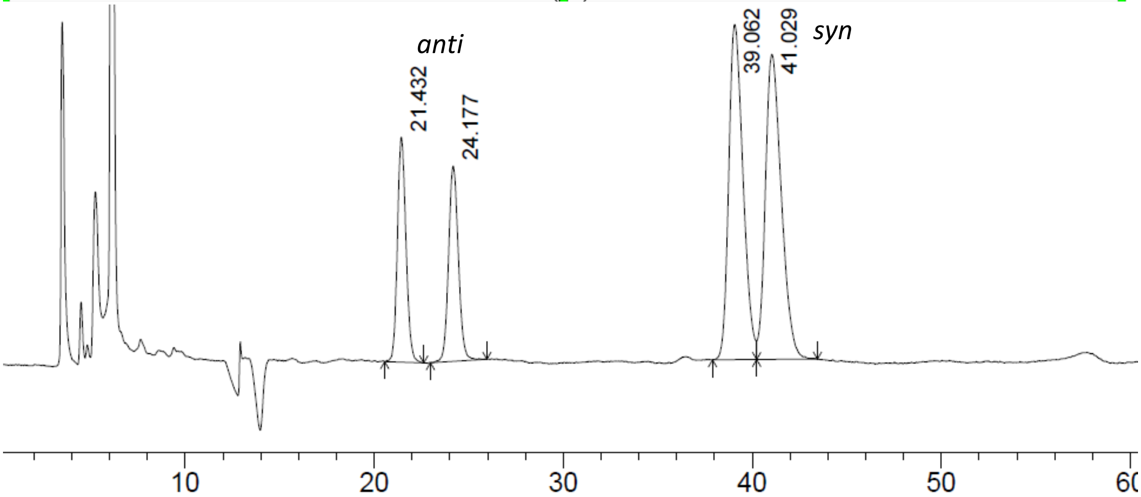

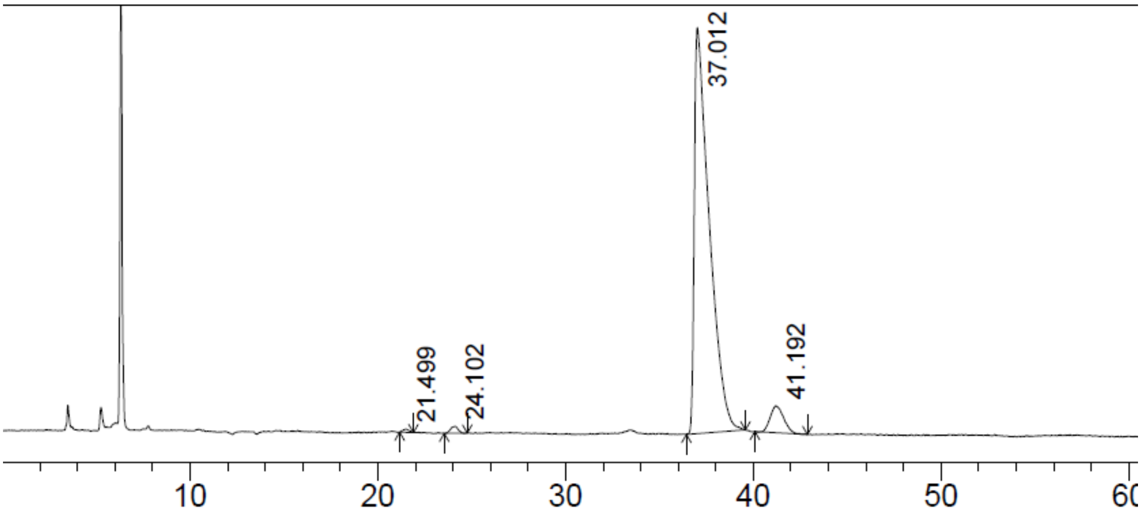

HPLC: ID column, 1 ml/min, hexane:isopropanol 95:5, 209 nm

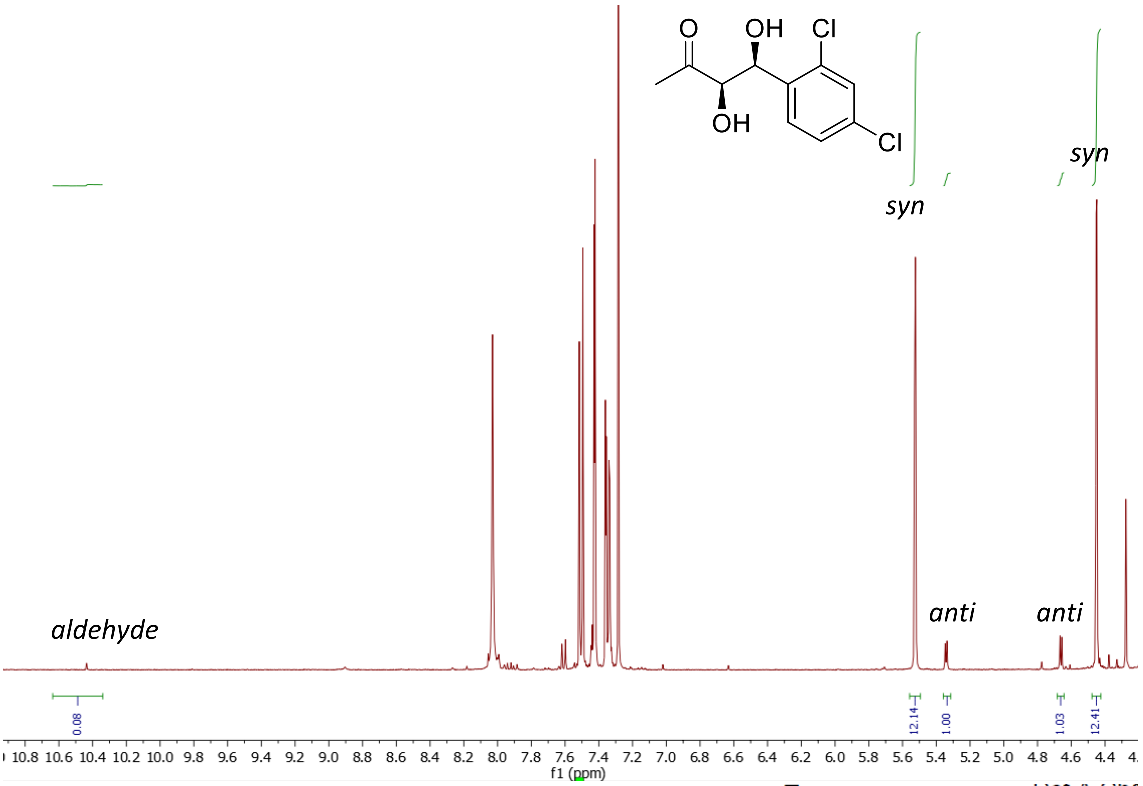

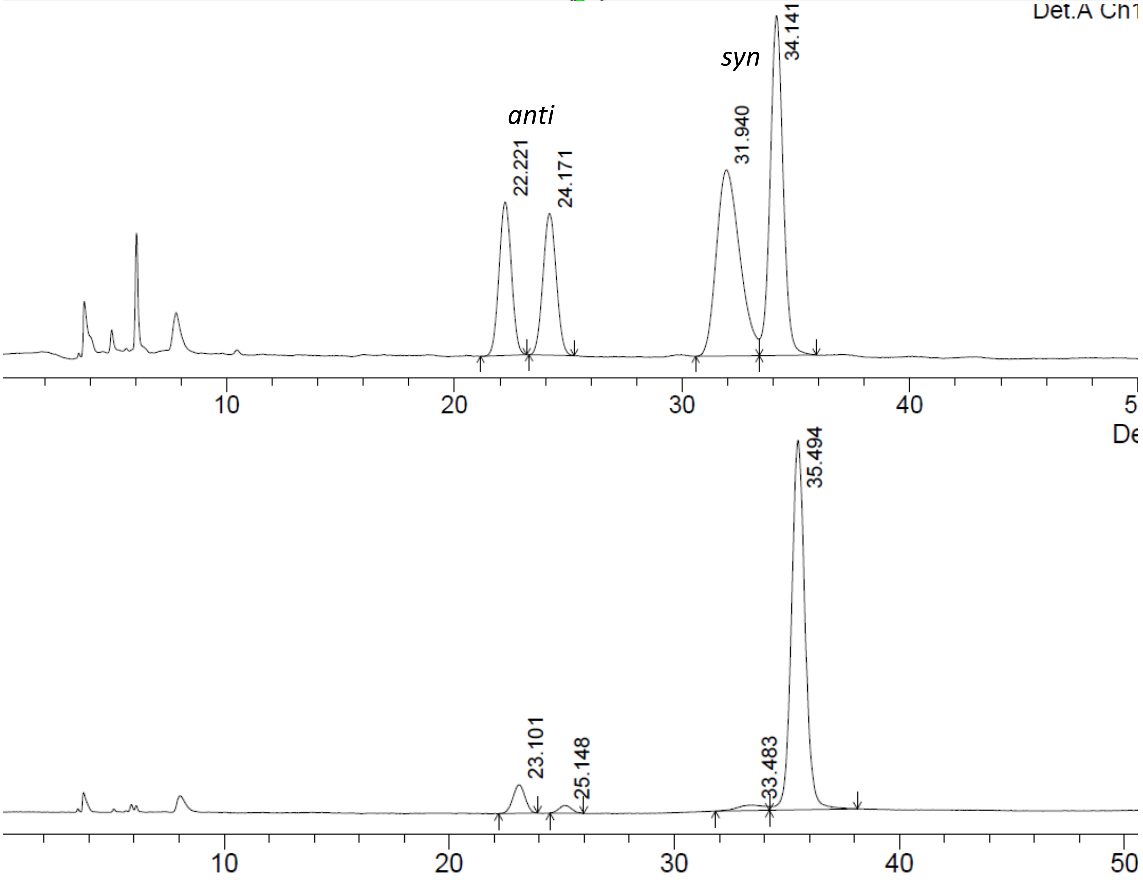

HPLC: IC column, 1 ml/min, hexane:isopropanol 98:2, 209 nm

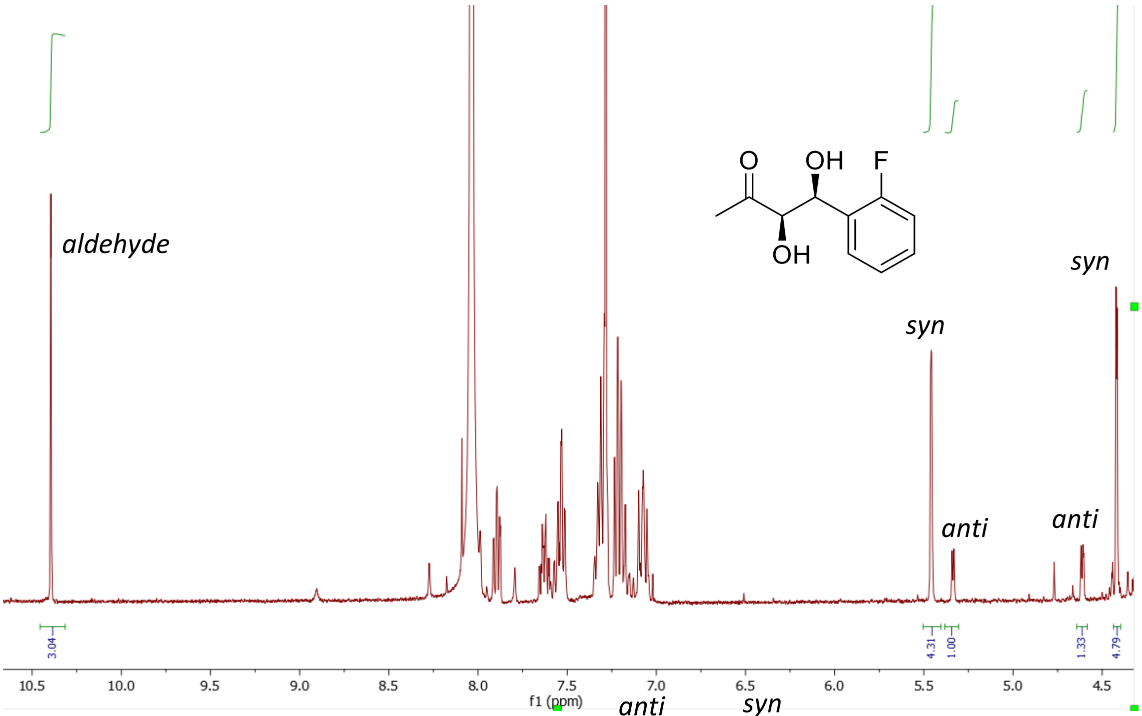

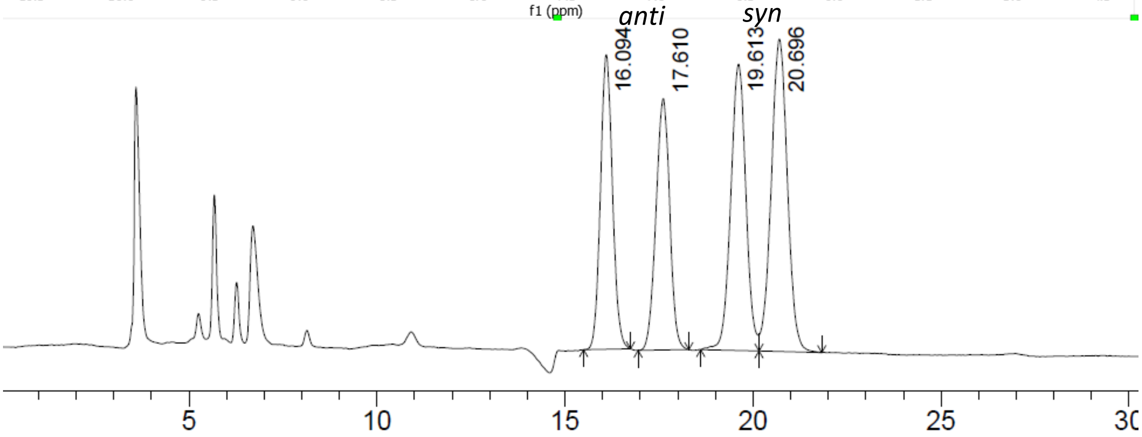

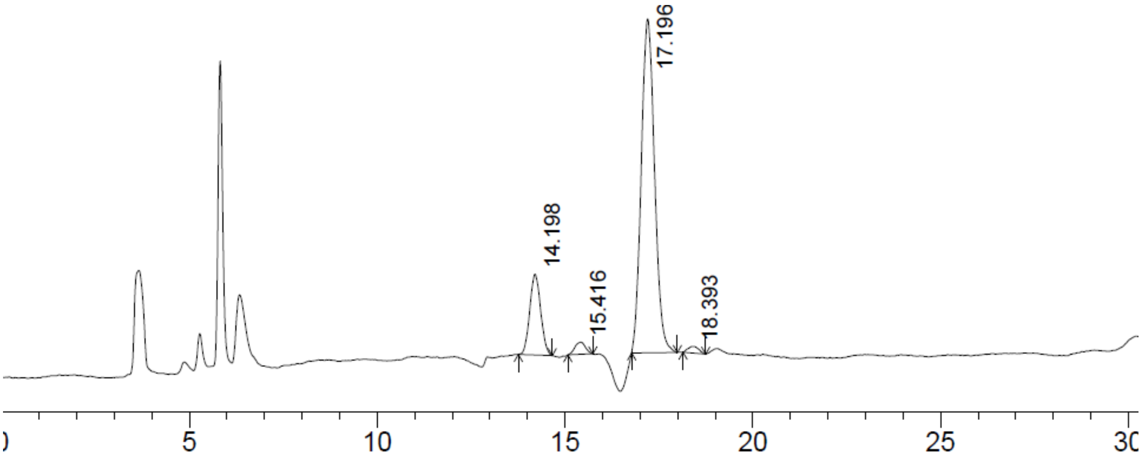

HPLC: IC column, 1 ml/min, hexane:isopropanol 95:5, 209 nm

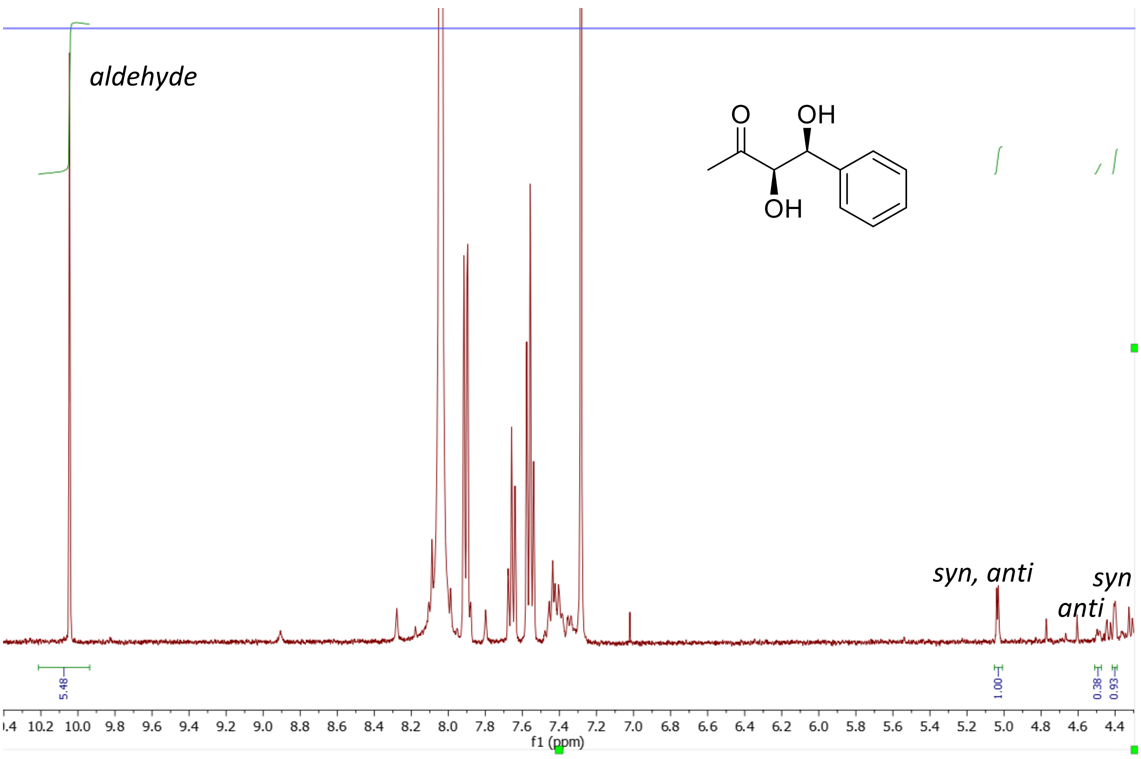

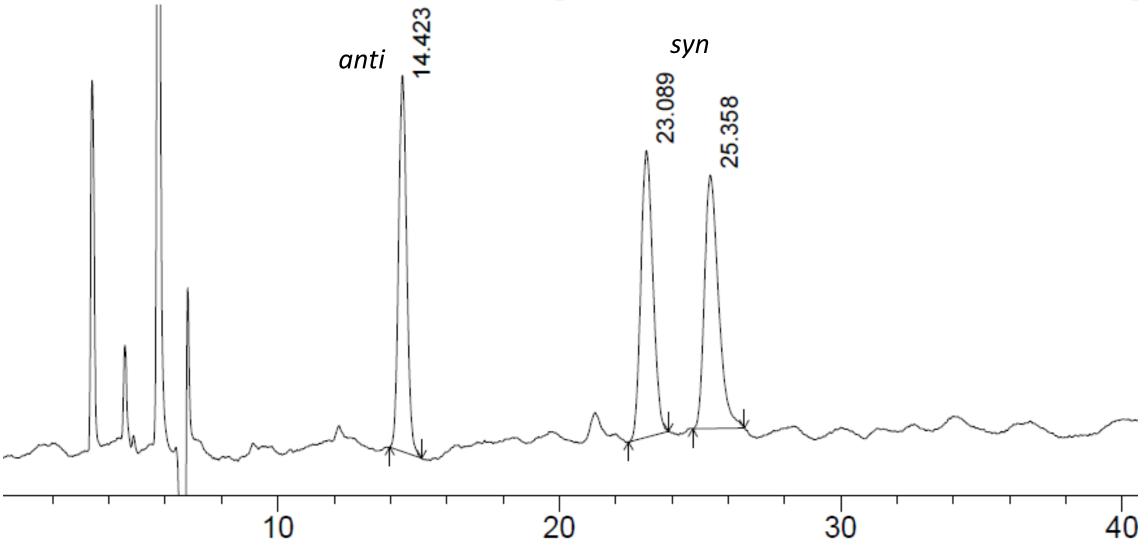

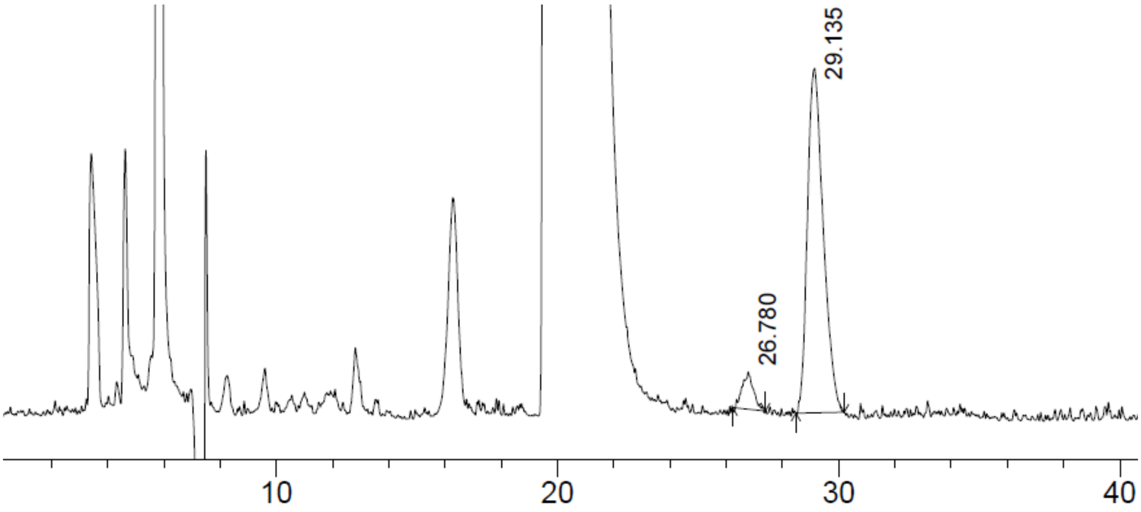

HPLC: ID column, 1 ml/min, hexane:isopropanol 90:10, 209 nm
